# Significance of Serum Pepsinogens as a Biomarker for Gastric Cancer and Atrophic Gastritis Screening: A Systematic Review and Meta-Analysis

**DOI:** 10.1371/journal.pone.0142080

**Published:** 2015-11-10

**Authors:** Ya-kai Huang, Jian-chun Yu, Wei-ming Kang, Zhi-qiang Ma, Xin Ye, Shu-bo Tian, Chao Yan

**Affiliations:** Department of General Surgery, Peking Union Medical College Hospital, Chinese Academy of Medical Sciences and Peking Union Medical College, Beijing, China; Sapporo Medical University, JAPAN

## Abstract

**Background:**

Human pepsinogens are considered promising serological biomarkers for the screening of atrophic gastritis (AG) and gastric cancer (GC). However, there has been controversy in the literature with respect to the validity of serum pepsinogen (SPG) for the detection of GC and AG. Consequently, we conducted a systematic review and meta-analysis to assess the diagnostic accuracy of SPG in GC and AG detection.

**Methods:**

We searched PubMed, Embase, and the Chinese National Knowledge Infrastructure (CNKI) for correlative original studies published up to September 30, 2014. The summary sensitivity, specificity, positive diagnostic likelihood ratio (DLR+), negative diagnostic likelihood ratio (DLR-), area under the summary receiver operating characteristic curve (AUC) and diagnostic odds ratio (DOR) were used to evaluate SPG in GC and AG screening based on bivariate random effects models. The inter-study heterogeneity was evaluated by the I^2^ statistics and publication bias was assessed using Begg and Mazumdar’s test. Meta-regression and subgroup analyses were performed to explore study heterogeneity.

**Results:**

In total, 31 studies involving 1,520 GC patients and 2,265 AG patients were included in the meta-analysis. The summary sensitivity, specificity, DLR+, DLR-, AUC and DOR for GC screening using SPG were 0.69 (95% CI: 0.60–0.76), 0.73 (95% CI: 0.62–0.82), 2.57 (95% CI: 1.82–3.62), and 0.43 (95% CI: 0.34–0.54), 0.76 (95% CI: 0.72–0.80) and 6.01 (95% CI: 3.69–9.79), respectively. For AG screening, the summary sensitivity, specificity, DLR+, DLR-, AUC and DOR were 0.69 (95% CI: 0.55–0.80), 0.88 (95% CI: 0.77–0.94), 5.80 (95% CI: 3.06–10.99), and 0.35 (95% CI: 0.24–0.51), 0.85 (95% CI: 0.82–0.88) and 16.50 (95% CI: 8.18–33.28), respectively. In subgroup analysis, the use of combination of concentration of PGI and the ratio of PGI:PGII as measurement of SPG for GC screening yielded sensitivity of 0.70 (95% CI: 0.66–0.75), specificity of 0.79 (95% CI: 0.79–0.80), DOR of 6.92 (95% CI: 4.36–11.00), and AUC of 0.78 (95% CI: 0.72–0.81), while the use of concentration of PGI yielded sensitivity of 0.55 (95% CI: 0.51–0.60), specificity of 0.79 (95% CI: 0.76–0.82), DOR of 6.88 (95% CI: 2.30–20.60), and AUC of 0.77 (95% CI: 0.73–0.92). For AG screening, the use of ratio of PGI:PGII as measurement of SPG yielded sensitivity of 0.69 (95% CI: 0.52–0.83), specificity of 0.84 (95% CI: 0.68–0.93), DOR of 11.51 (95% CI: 6.14–21.56), and AUC of 0.83 (95% CI: 0.80–0.86), the use of combination of concentration of PGI and the ratio of PGI:PGII yield sensitivity of 0.79 (95% CI: 0.72–0.85), specificity of 0.89 (95% CI: 0.85–0.93), DOR of 24.64 (95% CI: 6.95–87.37), and AUC of 0.87 (95% CI: 0.81–0.92), concurrently, the use of concentration of PGI yield sensitivity of 0.46 (95% CI: 0.38–0.54), specificity of 0.93 (95% CI: 0.91–0.95), DOR of 19.86 (95% CI: 0.86–456.91), and AUC of 0.86 (95% CI: 0.52–1.00).

**Conclusion:**

SPG has great potential as a noninvasive, population-based screening tool in GC and AG screening. In addition, given the potential publication bias and high heterogeneity of the included studies, further high quality studies are required in the future.

## Introduction

Gastric cancer (GC) was the fifth most common cancer and the third leading cause of cancer-related mortality worldwide [1]. In the Asia-Pacific region, the incidence of GC is high in Japan, China, Korea, Singapore, and Malaysia and is low in Thailand, India, New Zealand and Australia[2]. The clinical symptoms in the early stages of GC are not specific; therefore, a large number of patients with early GC do not seek appropriate medical care until the disease has progressed [3], and the prognosis of patients with advanced GC remains poor [4]. GC develops in a stepwise manner, and subjects with precancerous lesions, such as atrophic gastritis (AG), intestinal metaplasia (IM), and dysplasia, may be at high risk of eventually developing carcinoma. Subsequently, it is important to improve the prognosis of GC by identifying its high-risk population. The development of tools for the early diagnosis of GC and precancerous lesions of GC is important for reducing mortality, increasing survival rates, and improving quality of life[5]. Endoscopy and biopsy are the reference standards for diagnosis and screening of GC and precancerous lesions of GC, but their use is limited for population-wide screening due to their invasiveness[6, 7]. Subsequently, it is necessary to identify novel, simple, cost-effective and manipulable screening methods for GC and precancerous lesions of GC.

Human pepsinogens are proenzymes for pepsin, a digestive enzyme produced by gastric chief cells. Human pepsinogens are biochemically and immunochemically classified into two groups: pepsinogen I (PGI) and pepsinogen II (PGII)[8, 9]. PGI is secreted by chief and mucous neck cells in the fundic glands, whereas PGII is also secreted by cells in the pyloric and Brunner’s glands[10]. PGI and II are secreted into the gastric lumen, and approximately 1% can be found in the serum. Serum pepsinogen (SPG) may function as a marker of the functional and morphologic status of the gastric mucosa, including atrophic changes and inflammation, such as *H*. *pylori* infection, AG and IM[11]. Serum PGI and PGII levels are increased with increasing severity of *H*. *pylori*-related chronic gastritis. However, when atrophic changes in the corpus are accompanied by a loss of cells in the corpus, including those secreting PGI, the level of PGI decreases, whereas the level of PGII remains high or stable. Therefore, the ratio of PGI:PGII decreases in a stepwise manner. More severe atrophy is related to a lower PGI:PGII ratio. The non-invasive markers PGI and PGII and their ratio have been proposed as predictors of various gastric pathologies, including AG and IM[10, 12], which are defined as precancerous lesions for GC[13]. In addition, several case-control and cohort studies have demonstrated the predictive value of SPG for GC diagnosis and screening, suggesting that it is possible to use SPG for GC screening on the basis of large populations. In Japan, SPG detection has become the first step of GC screening, instead of photofluorography[14].

SPG has been commonly accepted as a useful biomarker for GC screening and AG diagnosis, but its efficacy remains controversial. To obtain summary estimates of the diagnostic accuracy of SPG for screening GC and for the diagnosis of AG, the present meta-analysis was performed to assess the overall diagnostic performance of SPG in patients with GC or AG.

## Materials and methods

### Search strategy

Electronic searches were performed using PubMed, Embase, and the Chinese National Knowledge Infrastructure (CNKI). To assess the diagnostic value of SPG in GC, the following search terms were used: (1) (((((pepsinogen[Title/Abstract]) AND gastric cancer[Title/Abstract])) OR ((pepsinogen[Title/Abstract]) AND gastric carcinoma[Title/Abstract])) OR ((pepsinogen[Title/Abstract]) AND stomach carcinoma[Title/Abstract])) OR ((pepsinogen[Title/Abstract]) AND stomach cancer[Title/Abstract]) in PubMed and CNKI; (2) (TITLE-ABSTR-KEY(pepsinogen) and TITLE-ABSTR-KEY(stomach cancer)) or (((TITLE-ABSTR-KEY(pepsinogen) and TITLE-ABSTR-KEY(gastric cancer)) or (TITLE-ABSTR-KEY(pepsinogen) and TITLE-ABSTR-KEY(gastric carcinoma))) or (TITLE-ABSTR-KEY(pepsinogen) and TITLE-ABSTR-KEY(stomach carcinoma))) in Embase (ScienceDirect). The search terms used for AG diagnosis by SPG were presented as follows: (1) ((((gastritis[Title/Abstract]) AND pepsinogen[Title/Abstract])) OR ((intestinal metaplasia [Title/Abstract]) AND pepsinogen[Title/Abstract])) OR ((dysplasia[Title/Abstract]) AND pepsinogen[Title/Abstract]) in PubMed and CNKI; (2) ((TITLE-ABSTR-KEY(pepsinogen) and TITLE-ABSTR-KEY(gastritis)) or (TITLE-ABSTR-KEY(pepsinogen) and TITLE-ABSTR-KEY(intestinal metaplasia))) or (TITLE-ABSTR-KEY(pepsinogen) and TITLE-ABSTR-KEY(dysplasia)) in Embase (ScienceDirect). The reference lists of all retrieved articles were reviewed to identify additional potentially relevant studies in adherence with the preferred reporting items for systematic reviews and meta-analysis (PRISMA) guidelines.

### Selection criteria

The studies included in the meta-analysis met the following criteria: (1) the studies or article abstracts were written in English or Chinese; (2) all GC, AG, IM or dysplasia patients were histologically confirmed by pathologists; (3) the studies detected serum or plasma pepsinogen in GC, AG, IM or dysplasia; (4) peripheral blood was collected for SPG detection before treatment; and (5) the study presented sensitivity, specificity, and clear cut-off values. If the articles contained the same or overlapping data, the largest or most recent populations were selected. Unqualified data, duplicate publications, case reports, conference abstracts, reviews, letters to journal editors, articles with no clear cut-off value, and small scale studies with fewer than 30 cases were excluded. The retrieved studies were evaluated, and relevant data from the included studies were extracted independently by two investigators (YK.H. and SB.T.). Disagreement was resolved by discussion.

### Data extraction and quality assessment of studies

The data extracted from studies included the following: (1) basic characteristics of studies, including: first author’s name; publication year; country of origin; the number of patients and controls; detection method; cut-off values; study design; pathological type and mean age; and (2) diagnostic performance, including sensitivity, specificity, TP, FP, FN, and TN. The Quality Assessment of Diagnostic Accuracy Studies-2 (QUADAS-2) checklist was used by two reviewers (C.Y. and YK.H.) to assess study quality using RevMan 5.3[15]. However, studies were not excluded on the basis of quality. A proportional bar graph and summary table of review authors' ratings for each criterion was plotted to characterize the results of our assessment.

### Statistical analysis

We calculated the pooled sensitivity, pooled specificity, diagnostic odds ratio (DOR), positive diagnostic likelihood ratio (DLR+), negative diagnostic likelihood ratio (DLR-), and 95% confidence intervals (CIs) for each criterion. A summary receiver operator characteristic (SROC) curve was generated, and the area under the summary receiver operating characteristic curve (AUC) was calculated[16]. To assess the clinical utility of SPG for GC and AG diagnosis, Fagan’s nomograms were plotted. The threshold effect was assessed by Spearman’s correlation analysis. Heterogeneity was assessed using the I^2^ statistics; I^2^>50% indicated moderate to high heterogeneity[17]. Meta-regression was performed to identify possible sources of heterogeneity. Subgroup analyses were also performed as necessary. Sensitivity analysis was performed to evaluate the effects of each individual study on the summary accuracy of SPG detection for GC and AG. A funnel plot followed by the Begg and Mazumdar's test was used to explore potential publication bias. All analyses were performed with Stata 12.0 (College Station, TX, USA), Meta-DiSc statistical software[18], and RevMan 5.3 (Cochrane, USA).

## Results

### Identification of studies

**Fig 1:**
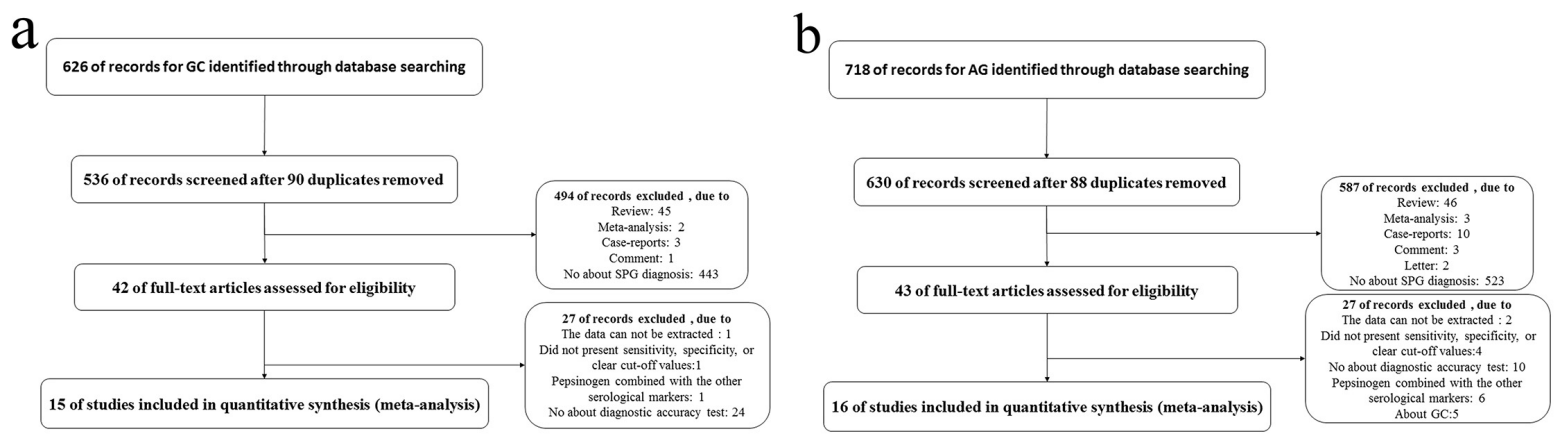
Flow chart of the included studies. (a) Flow chart for GC; (b) Flow chart for AG.

For GC diagnosis, our initial database search retrieved 626 published articles, 90 of which were duplicates and were excluded. Among the remaining studies, 443 articles were not relevant to our research topic, 47 were meta-analyses or reviews, and 4 were comments or case-reports. Finally, 42 articles were subject to full-text analysis. One of these articles was excluded because the data could not be extracted, 1 were excluded because the studies did not present sensitivity, specificity, or clear cut-off values, 1 was excluded because of the combination of SPG and other serological markers, and 24 were excluded for not including a diagnostic accuracy test. Ultimately, 15 eligible articles were included in the present meta-analysis [6, 19–32](Fig 1A). For AG, IM or dysplasia diagnoses, our initial database search retrieved 718 published articles, 88 of which were duplicates and were excluded. Among the remaining studies, 523 articles did not meet the criteria, 49 were meta-analyses or reviews, and 15 were comments, letters or case-reports. Finally, 43 articles were subject to full-text analysis. Two of these articles were discarded because the data could not be extracted, 4 were discarded the studies did not present sensitivity, specificity, or clear cut-off values, 10 were discarded for having no correlation to the diagnostic accuracy test, 6 were discarded because of the combination of SPG and other serological markers, and 5 were discarded due to the application of SPG in GC diagnosis. Sixteen eligible articles were included in the present meta-analysis [12, 31, 33–46] (Fig 1B). No unpublished relevant studies were obtained.

### Study characteristics and quality assessment

The characteristics of the included studies are summarized in Table 1. Briefly, the 31 studies represented 13 countries. In total, 27 studies were published in English, 2 were written in Chinese, and 1 was written in Korean. Overall, 1,520 GC patients and 27,723 control samples were included in 15 studies with respect to GC diagnosis. A total of 2,265 AG patients and 2,660 control samples were included in the 16 studies with respect to the diagnosis of AG. All patients were diagnosed by endoscopy and biopsy. The studies were published from 1991 to 2014 and used different detection methods and cut-off values, although most involved radio-immunity assays (RIA) and enzyme-linked immunosorbent assays (ELISA). The most commonly used cut-off values were PG I≤70 ng/ml and PG I:PG II≤3.0 (Table 1). Four articles contained different cut-off values within the same study, and we selected the cut-off values with the highest Youden’s index for the present analysis. For GC diagnosis, the sensitivity and specificity ranges were 37–91% and 36–97%, respectively, and the sensitivity and specificity ranges for the diagnosis of AG using SPG were 17–91% and 39–100%, respectively.

**Table 1:** Characteristics of the studies included in the meta-analysis

Author/year/region	Patients (n)	Control (n)	Cut-off	SPG detection method	Pathological type	TP (n)	FP (n)	FN (n)	TN (n)	Design	Mean age of patients (years)*
F Kitahara, 1999, Japan[19]	13	5,100	PGI<70 ng/ml and PGI:PGII<3.0	RIA	GC (cardia/non cardia: 1/12)	11	1352	2	3,748	Cohort	52.3
Abraham M. Y. Nomura, 2005, Hawaii[20]	250	334	PGI≤30 ng/ml	RIA	GC (cardia/non cardia: 29/221)	96	65	154	169	Case-control	70.7
Masahira Haneda, 2012, Japan[21]	47	214	PGI:PGII≤4.5	CLIA	GC	26	62	21	152	Case-control	57
Ryousuke Kikuchi, 2011, Japan[22]	122	179	PGI≤30 ng/ml and PGI:PGII≤2.0	CLIA	GC	95	68	27	109	Case-control	68.2
KENTARO SHIKATA, 2012, Japan[6]	69	2,377	PGI≤59 ng/ml and PGI:PGII≤3.9	RIA	GC	49	731	20	1,646	Cohort	57.3
Rafael Lomba-Viana, 2012, Portugal[23]	9	505	PGI≤70 ng/ml and PGI:PGII≤3.0	ELISA	GC (cardia/non cardia: 0/9)	6	268	3	237	Cohort	64
Jung Mook Kang, 2008, South Korea[24]	380	686	PGI:PGII≤3.0	L-TIA	GC	225	268	155	418	Case-control	57.6
Xiao-mei Zhang, 2014, China[25]	82	142	PGI<70 ng/ml	ELISA	GC	56	25	26	117	Case-control	NR
SHIGETO MIZUNO, 2009, Japan[26]	19	12,101	PGI≤30 ng/ml and PGI:PGII≤2.0	CLIA	GC (cardia/non cardia: 1/18)	7	486	12	11,615	Cohort	57
Yu-Yan Huang, 2013, China[27]	55	211	PGI≤73.14 ng/ml	ELISA	GC	50	58	5	153	Case-control	60
Zhong-Lin Yu, 2008, China[28]	148	2,520	PGI≤70 ng/ml and PGI:PGII≤3.0	ELISA	GC	28	96	27	443	Cohort	NR
Metin Agkoc, 2010, Turkey[31]	50	30	PGI<25 ng/ml and PGI:PGII<3.0	RIA	GC	41	1	9	29	Case-control	65.42
Masaharu Yoshihara, 1998, Japan[29]	25	3157	PGI≤50 ng/ml and PGI:PGII≤3.0	RIA	GC	21	2028	4	1129	Cohort	Null
F.-Y. CHANG, 1992, Taiwan[30]	192	70	PGI<70 ng/ml	RIA	GC	124	12	68	58	Case-control	Null
Kazuo Aoki,1997, japan[32]	59	97	PG I<40 ng/ml and PG I:PG II<3.0	RIA	GC	38	13	21	84	Case-control	64
A. Oksanen, 2000, Finland[33]	90	54	PGI<84.4 ng/ml	ELISA	AG	15	0	75	54	Cohort	55
Cai-yun He, 2011, China[34]	1,556	466	PGIFF1E8.25 ng/ml	ELISA	AG	1099	136	457	330	Cohort	53.16
Diana Aulia, 2009, Indonesia[35]	26	37	PGI<119 ng/ml	ELISA	AG	18	18	8	19	Case-control	47.6
Metin Agkoc, 2010, Turkey[31]	30	30	PGI<25 ng/ml and PGI:PGII<3.0	RIA	AG	27	0	3	30	Case-control	32.86
Katsunori Iijima, 2009, Japan[36]	20	142	PGI≤70 ng/ml and PGI:PGII≤2.0	ELISA	AG	9	6	11	136	Cohort	55
Hyojin Chae, 2008, Korea[37]	59	67	PGI:PGII<4.0	L-TIA	AG	49	6	10	61	Cohort	50.8
R. Sierra, 2006, Costa Rica[38]	34	400	PGI:PGII<3.4	ELISA	AG	31	246	3	154	Cohort	46
Ma ´rio Dinis-Ribeiro, 2004, Portugal[39]	61	74	PGI:PGII<3.05	ELISA	AG with IM	40	16	21	58	Cohort	61
Kai Chun WU, 2004, China[40]	27	54	PGI:PGII<8.1	ELISA	AG	24	9	3	45	Cohort	64.8
N Broutet, 2003, Finland[41]	62	222	PGI:PGII<5.6	RIA	AG	40	49	22	173	Cohort	43.5
Abbas Zoalfaghari, 2013, Iran [42]	51	59	PGI:PGII<4.0	ELISA	AG	36	17	15	42	Cohort	51.4
David Y Graham, 2006 Mexico [12]	5	122	PGI:PGII<6.7	ELISA	AG	4	35	1	87	Cohort	NR
M. Kekki, 1991, Finland [43]	46	654	PGI<30 ng/ml	RIA	AG	41	34	5	620	Cohort	47
G. Nardone, 2005, Italy [44]	30	64	PGI:PGII<3.0	ELISA	AG	9	0	21	64	Cohort	56
Manami Inoue, 1998, japan [45]	117	83	PGI≤70 ng/ml and PGI:PGII≤3.0	RIA	AG	96	21	21	62	Cohort	60.5
F. Sitas, Netherlands, 1993 [46]	28	33	PGI:PGII<1.5	ELISA	AG	7	2	21	31	Case-control	47.4

Note: RIA, radio-immunity assay; ELISA, enzyme-linked immunosorbent assay; CLIA, chemiluminescent immunoassay; L-TIA, latex-enhanced turbidimetric immunoassay; NR, no report.

*mean age of patients; GC, gastric cancer; AG, atrophic gastritis; IM, intestinal metaplasia.

**Fig 2:**
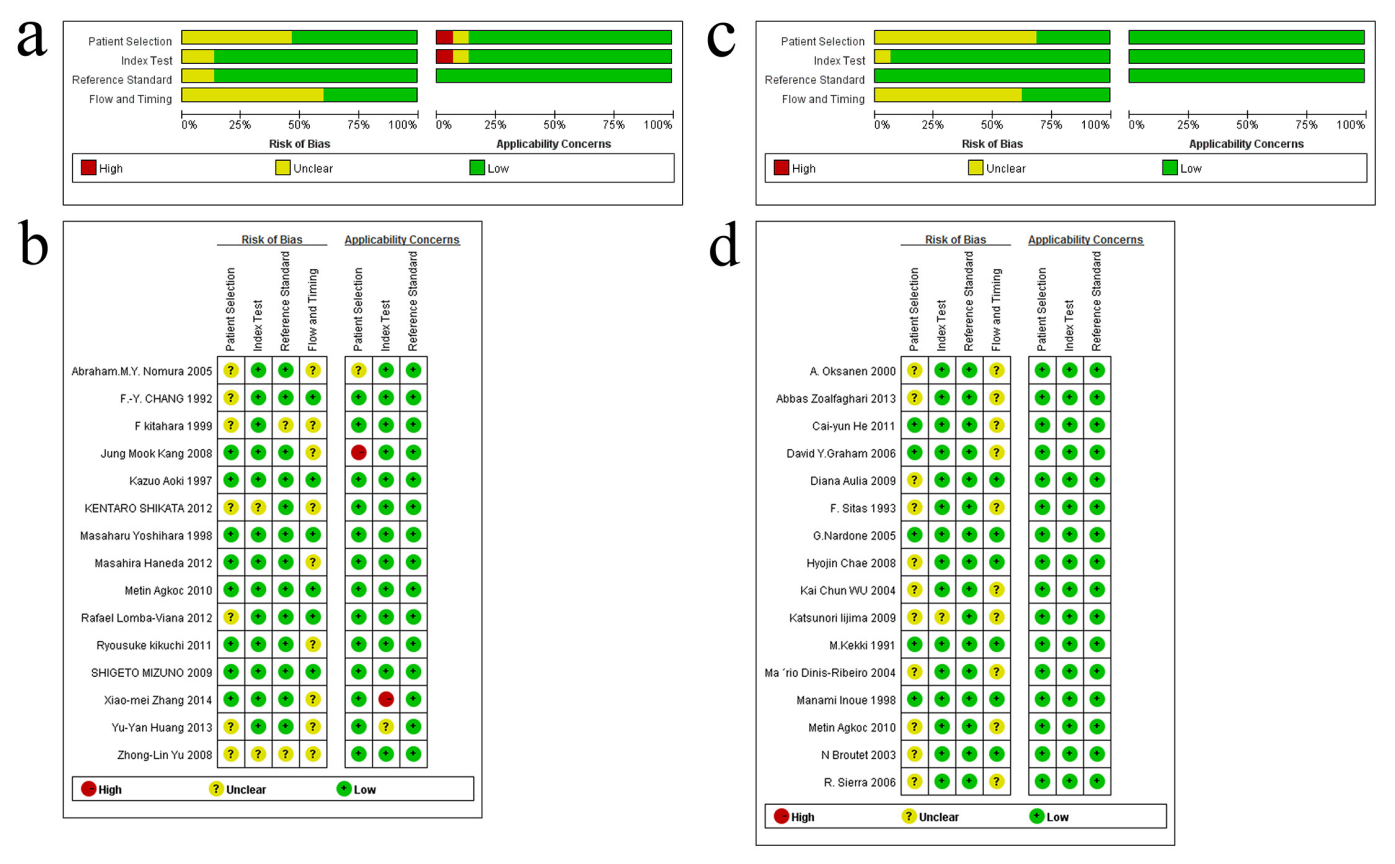
Quality assessment of the included studies using Quality Assessment of Diagnostic Accuracy Studies-2 (QUADAS-2) criteria. (a) Risk of bias and applicability concerns graph: review authors’ judgements about each domain presented as percentages across the included studies for GC; (b) Risk of bias and applicability concerns summary: review authors’ judgements about each domain for each included study for GC; (c) Risk of bias and applicability concerns graph: review authors’ judgements about each domain presented as percentages across the included studies for AG; (d) Risk of bias and applicability concerns summary: review authors’ judgements about each domain for each included study for AG.

An assessment of the studies by QUADAS-2 is presented in Fig 2. The overall quality of the eligible studies for GC diagnosis was not robust, but the studies showed good overall quality with respect to the diagnosis of AG. The index test and reference standard did not have an interaction effect for any of the included studies. For GC diagnosis, 6 of the 15 included studies had a cohort design, and 9 were case-control studies. For AG diagnosis, 13 of the 16 included studies had a cohort design, and 3 were case-control studies. For GC diagnosis, all studies had strict reference standards, and 9 contained clearly defined inclusion and exclusion criteria. Three of the 15 included studies did not employ an appropriate interval between the reference standard and the index test, which potentially led to the introduction of bias. For the diagnosis of AG, all included studies also had strict reference standards and employed an appropriate interval between the reference standard and the index test; 10 contained clearly defined inclusion and exclusion criteria, and 6 did not.

### Diagnostic accuracy of SPG in GC and AG

**Fig 3:**
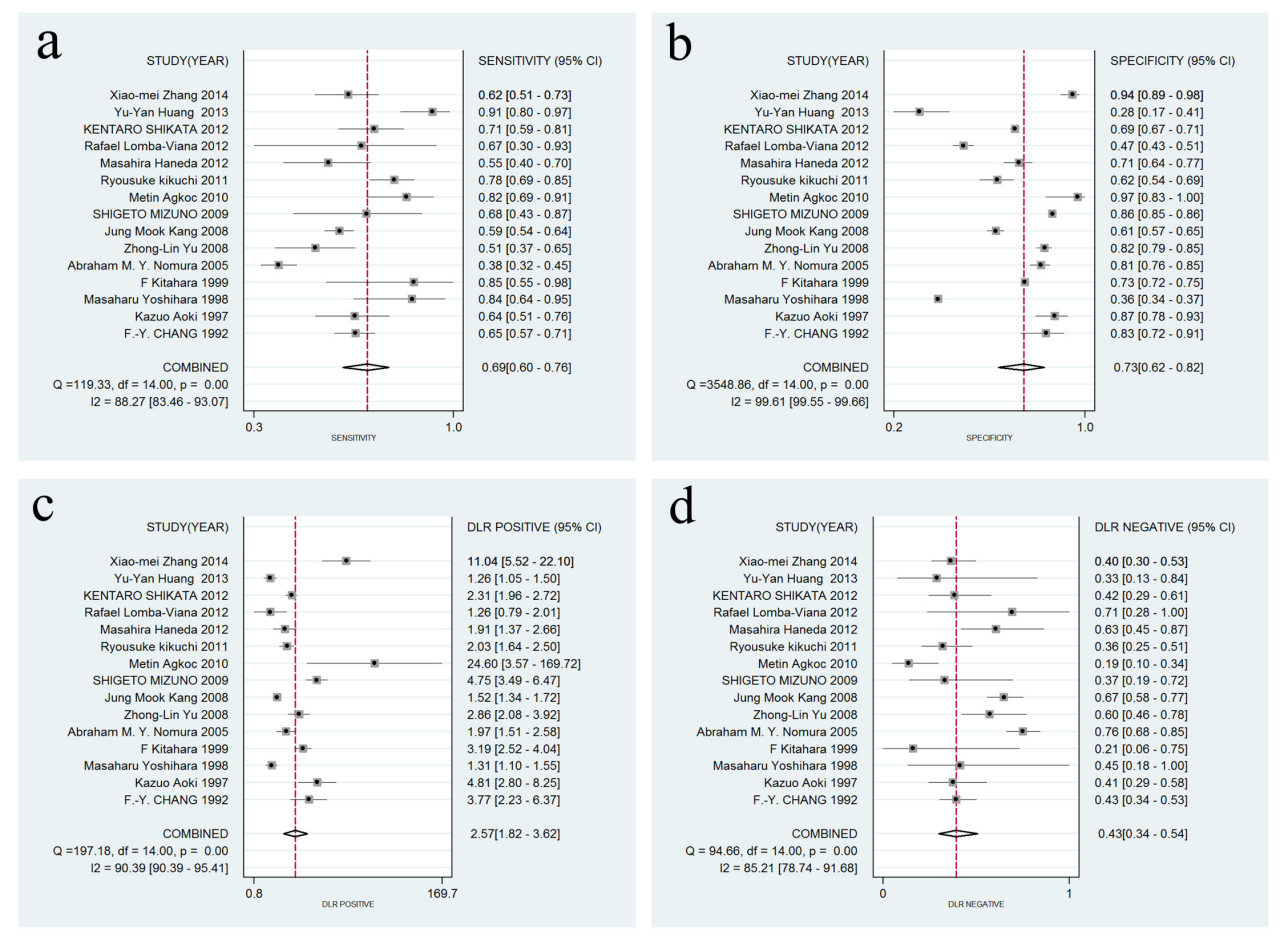
Forest plots of sensitivity, specificity, DLR+, and DLR- for SPG detection in GC. (a) The summary sensitivity was 0.69 (95% CI: 0.60–0.76; I2 = 88.27%; n = 15); (b) The summary specificity of all articles was 0.73 (95% CI: 0.62–0.82; I2 = 99.61%; n = 15); (c) The summary DLR+ of all articles was 2.57 (95% CI: 1.82–3.62; I2 = 90.39%; n = 15); (d) The summary DLR- of all articles was 0.43 (95% CI: 0.34–0.54; I2 = 85.21% n = 15).

**Fig 4:**
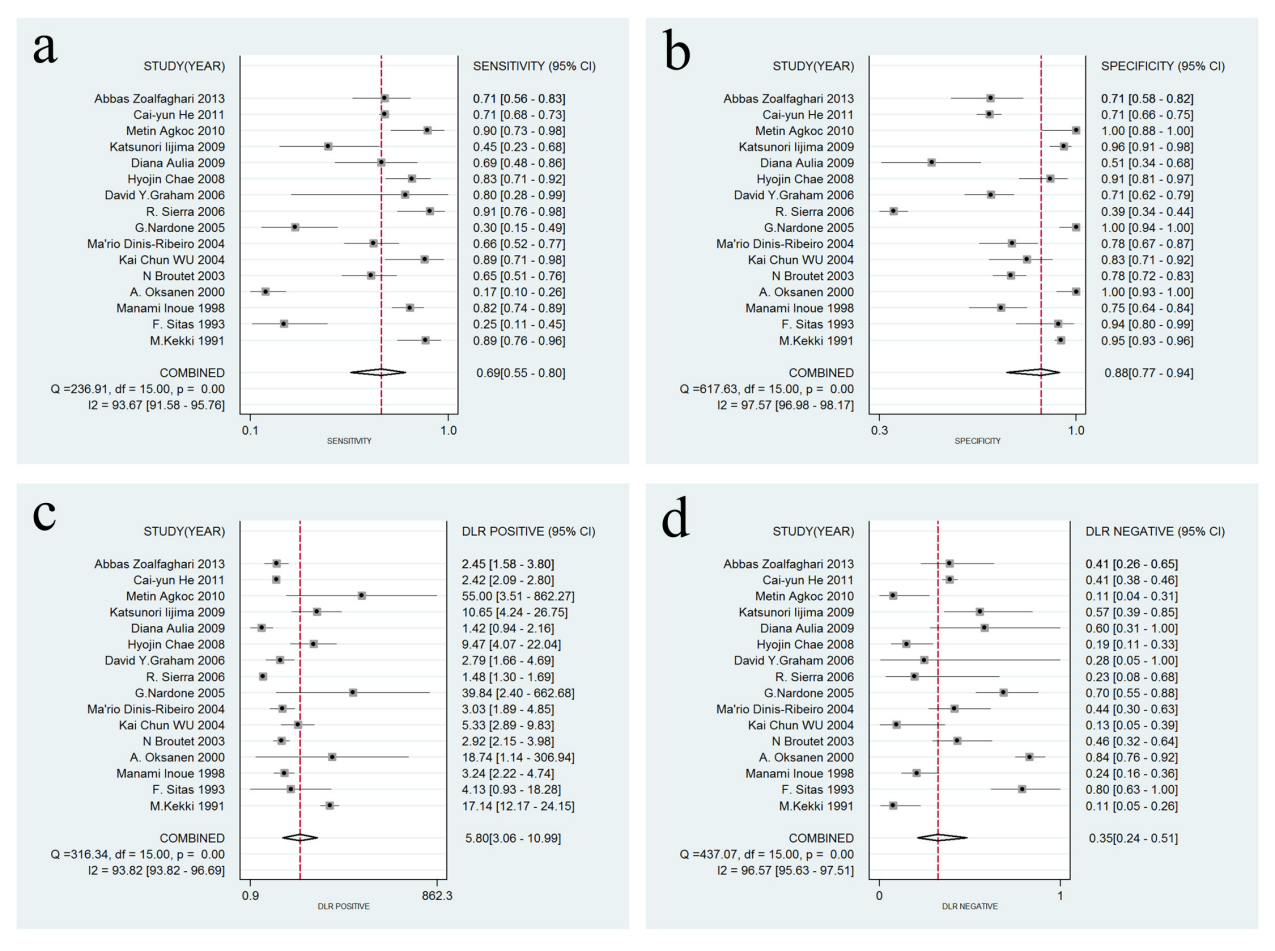
Forest plots of sensitivity, specificity, DLR+, and DLR- for SPG detection in AG. (a) The summary sensitivity was 0.69 (95% CI: 0.55–0.80; I2 = 93.67%; n = 16); (b) The summary specificity of all articles was 0.88 (95% CI: 0.77–0.94; I2 = 97.57%; n = 16); (c) The summary DLR+ of all articles was 5.80 (95% CI: 3.06–10.99; I2 = 93.82%; n = 16); (d) The summary DLR- of all articles was 0.35 (95% CI: 0.24–0.51; I2 = 96.57% n = 16).

**Fig 5:**
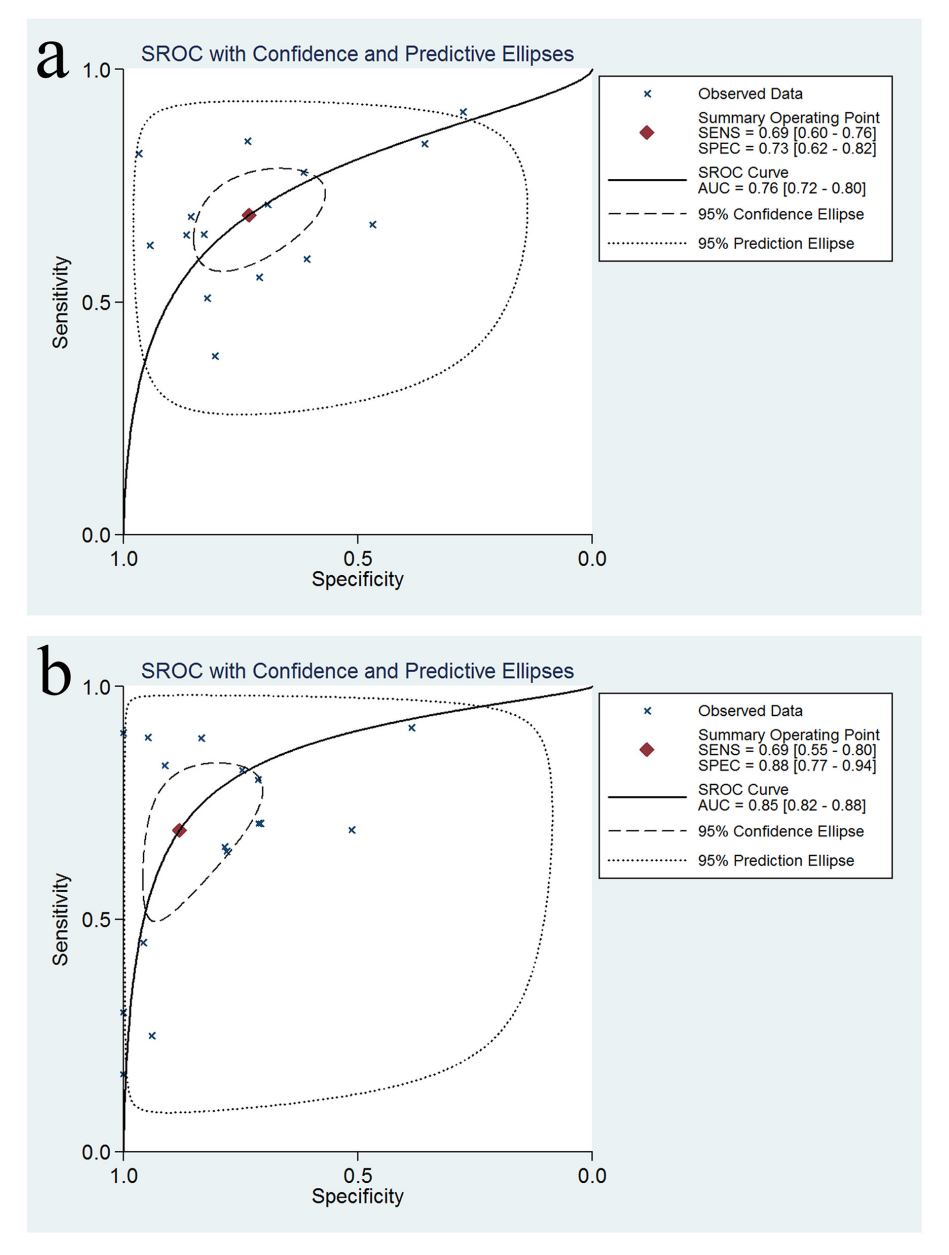
Summary ROC curve (SROC) with 95% confidence region and 95% prediction region. (a) SROC for SPG in the diagnosis of GC; (b) SROC for SPG in the diagnosis of AG.

**Fig 6:**
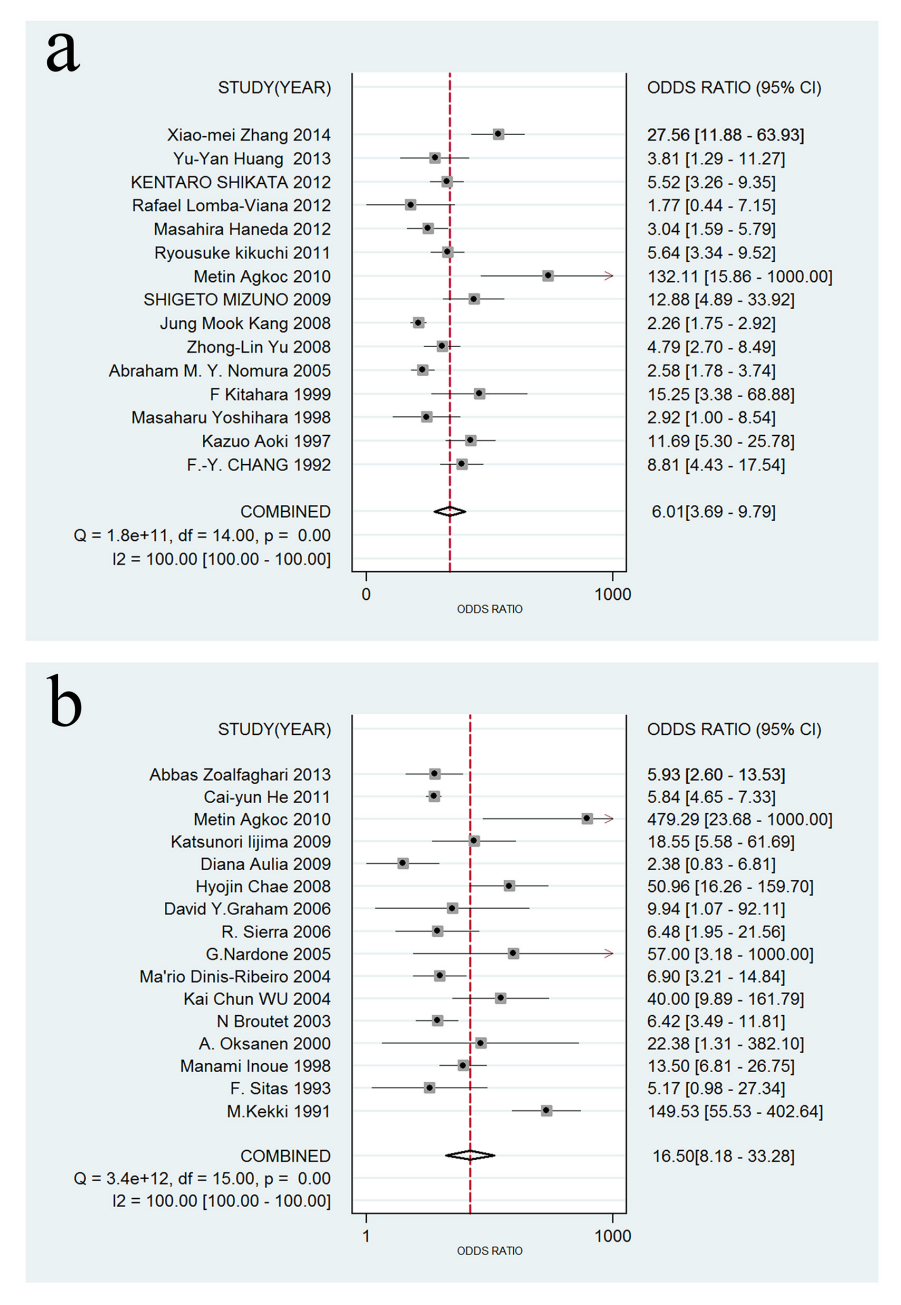
Forest plots of DOR for SPG detection in GC and AG. (a) For GC detection, the DOR was 6.01 (95% CI: 3.69–9.79); (b) For AG detection, the DOR was 16.50 (95% CI: 8.18–33.28).

**Fig 7:**
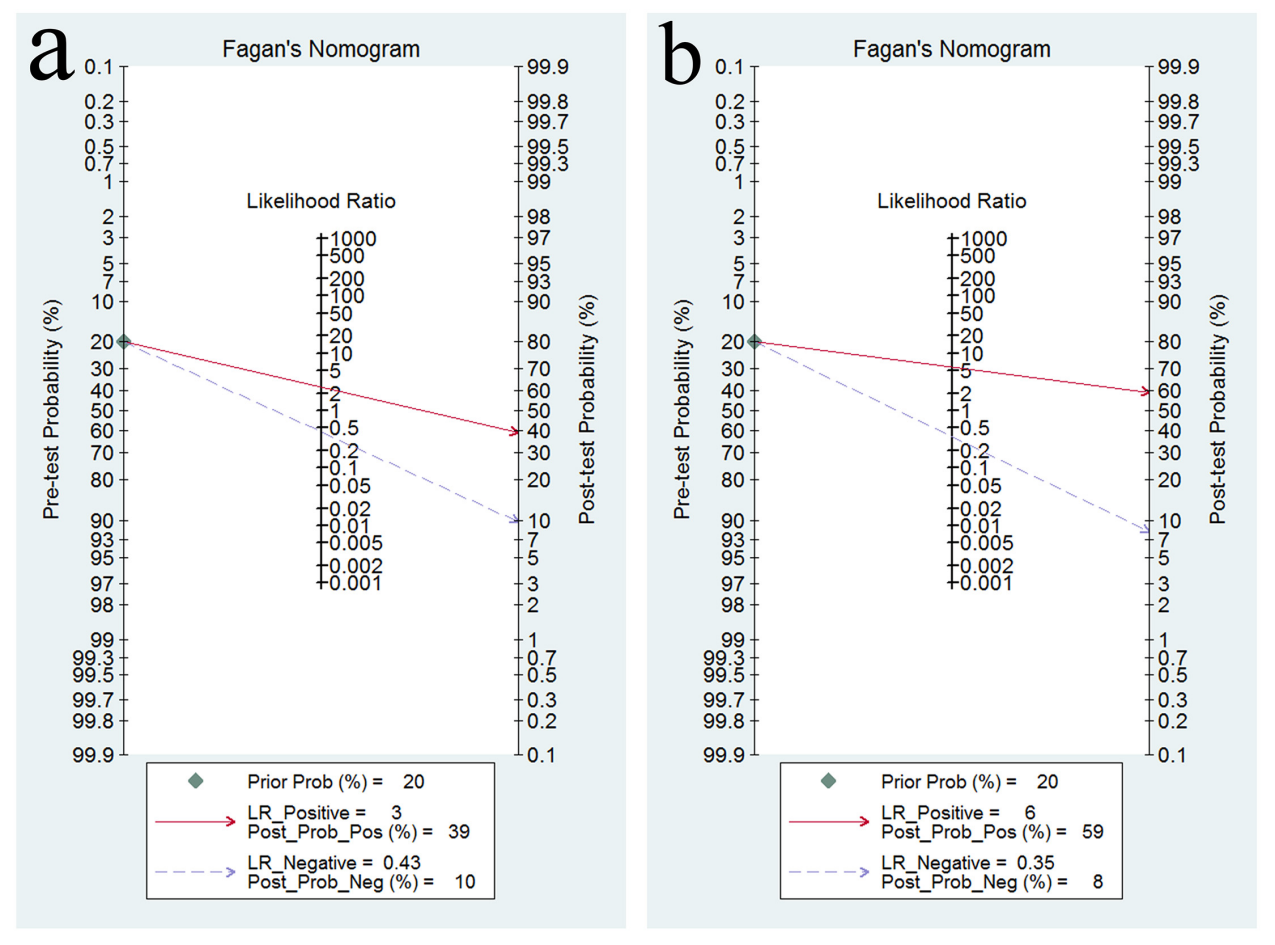
Fagan’s nomogram was plotted to calculate posterior probabilities. (a) Fagan plot for GC detection; (b) Fagan plot for AG detection.

A forest plot was used to demonstrate the sensitivity, specificity, DLR+, and DLR- for the detection of SPG in GC screening and AG diagnosis. The I^2^ values of the summary sensitivity, summary specificity, summary DLR+, and summary DLR- for studies of GC were 88.27% (95% CI: 83.46–93.07%), 99.61% (95% CI: 99.55–99.66%), 90.39% (95% CI: 90.39–95.41%), and 85.21% (95% CI: 78.74–91.68%), respectively. The I^2^ values of the summary sensitivity, summary specificity, summary DLR+, and summary DLR- for studies of AG were 93.67% (95% CI: 91.58–95.76%), 97.57% (95% CI: 96.98–98.17%), 93.82% (95% CI: 93.82–96.69%), and 96.57% (95% CI: 95.63–97.51%), respectively. The results indicated high heterogeneity in the sampled studies. Therefore, a random-effects model was performed. The resulting summary sensitivity, summary specificity, summary DLR+, and summary DLR- for studies of GC were 0.69 (95% CI: 0.60–0.76), 0.73 (95% CI: 0.62–0.82), 2.57 (95% CI: 1.82–3.62), and 0.43 (95% CI: 0.34–0.54) (Fig 3), respectively. The resulting summary sensitivity, summary specificity, summary DLR+, and summary DLR- for studies of AG were 0.69 (95% CI: 0.55–0.80), 0.88 (95% CI: 0.77–0.94), 5.80 (95% CI: 3.06–10.99), and 0.35 (95% CI: 0.24–0.51) (Fig 4), respectively. The SROC graphs with a 95% confidence region and a 95% prediction region are presented in Fig 5 and the forest plots of DOR are presented in Fig 6. For GC, the AUC was 0.76 (95% CI: 0.72–0.80), and the DOR was 6.01 (95% CI: 3.69–9.79). For AG, the AUC was 0.85 (95% CI: 0.82–0.88), and the DOR was 16.50 (95% CI: 8.18–33.28). In our study, we performed Spearman’s correlation analysis to explore possible threshold effects. Spearman’s correlation coefficient for GC was 0.486 (P = 0.066), and Spearman’s correlation coefficient for AG was 0.362 (P = 0.169), indicating no threshold effects. To assess the clinical utility of the index test, a Fagan’s nomogram was generated to compare the prior and posterior probabilities (Fig 7). For GC, when a prior probability of 20% was specified, the posterior probability positivity increased to 39%, with a DLR+ of 3.00. In addition, the posterior probability negativity decreased to 10.00%, with a DLR- of 0.43. A similar result was observed in AG diagnosis: when a prior probability of 20% was specified, the posterior probability positivity increased to 59%, with a DLR+ of 6.00, and the posterior probability negativity decreased to 8.00%, with a DLR- of 0.35. These findings suggest a moderate value for SPG in the diagnosis of GC and AG.

### Meta-regression and subgroup analysis

To explore the potential sources of inter-study heterogeneity, a meta-regression was performed for both GC and AG. The results indicated that the scale of the included patients could represent a potential source of heterogeneity for GC diagnosis (P = 0.0080), whereas the study design (P = 0.0295), SPG detection method (P = 0.0343) and measurement of SPG (P = 0.0334) were the major sources of heterogeneity for the SPG assay in AG detection. Consequently, we performed subgroup analyses, as shown in Table 2 and Table 3. For GC, the results indicated that studies with less than 50 patients exhibited an increased diagnostic accuracy of SPG detection compared with studies with greater than 50 patients. Similar findings were obtained in subgroups with the following characteristics: the use of ELISA method, the use of combination of concentration of PGI and the ratio of PGI:PGII as measurement of SPG, studies with appropriate interval between standard and index test, case-control design and studies not containing clearly defined inclusion and exclusion criteria. A latex-enhanced turbidimetric immunoassay (L-TIA) was commonly used to quantify serum proteins[47], and Huang M et al. established the use of reference intervals (RIs) for SPG in a healthy Chinese population using L-TIA[48]. We did not identify a sufficient number of studies to evaluate the diagnostic accuracy of the L-TIA test; therefore, we did not include the L-TIA in our subgroup analysis for GC. For AG, the diagnostic accuracy of SPG testing was higher in studies using combination of concentration of PGI and the ratio of PGI:PGII as the measurement of SPG than in studies with other measurements of SPG. Similar findings were also found in studies with cohort design, unclearly defined inclusion and exclusion criteria and the use of RIA method. The summary diagnostic accuracies of studies with the use of L-TIA method or concentration of PGII as measurement of SPG were not calculated because of an insufficient number of studies.

**Table 2:** Subgroup analysis of the included studies for GC.

Subgroup	N	Sensitivity (95% CI)	Specificity (95% CI)	DOR (95% CI)	AUC (95% CI)
Patient scale					
≤50 subjects	6	0.71 (0.64–0.78)	0.80 (0.80–0.81)	8.27 (5.06–13.51)	0.79 (0.69–0.88)
˃50 subjects	9	0.60 (0.57–063)	0.71 (0.70–0.72)	5.65 (3.48–9.17)	0.75 (0.68–0.79)
Detection method					
RIA	7	0.58 (0.54–0.62)	0.62 (0.61–0.63)	7.32 (3.74–14.33)	0.80 (0.69–0.89)
ELISA	3	0.73 (0.65–0.80)	0.61 (0.58–0.64)	11.99 (2.71–52.96)	0.84 (0.73–0.95)
CLIA	3	0.70 (0.63–0.77)	0.95 (0.95–0.96)	7.24 (4.50–11.65)	0.78 (0.75–0.80)
L-TIA	2	Null	Null	Null	Null
Measurement of SPG					
Combination of concentration of PGI and the ratio of PGI:PGII	9	0.70 (0.66–0.75)	0.79 (0.79–0.80)	6.92 (4.36–11.00)	0.78 (0.72–0.81)
Ratio of PGI:PGII	2	Null	Null	Null	Null
Concentration of PGI	4	0.55 (0.51–0.60)	0.79 (0.76–0.82)	6.88 (2.30–20.60)	0.77 (0.73–0.92)
Interval between standard and index test					
appropriate interval	12	0.61 (0.58–0.64)	0.62 (0.61–0.63)	6.53 (4.02–10.62)	0.77 (0.71–0.83)
Inappropriate interval	3	0.51 (0.42–0.57)	0.90 (0.90–0.91)	5.73(2.23–14.76)	0.75 (0.68–0.81)
Study design					
Cohort	6	0.64 (0.57–0.71)	0.79 (0.79–0.80)	5.54 (3.35–9.18)	0.74 (0.70–0.79)
Case-control	9	0.61 (0.58–0.63)	0.71 (0.69–0.73)	7.04 (3.90–12.69)	0.77 (0.68–0.86)
Clearly defined inclusion and exclusion criteria					
Yes	9	0.60 (0.57–0.63)	0.89 (0.89–0.90)	5.96 (3.59–9.88)	0.75 (0.68–0.82)
No	6	0.69 (0.62–0.75)	0.60 (0.59–0.61)	7.36 (3.20–16.93)	0.79 (0.68–0.90)

Note: AUC, area under the summary receiver operating characteristic curve; DOR, diagnostic odds ratio; RIA, radio-immunity assay; ELISA, enzyme-linked immunosorbent assay; CLIA, chemiluminescent immunoassay; L-TIA, latex-enhanced turbidimetric immunoassay; CI, confidence interval

**Table 3:** Subgroup analysis of the included studies for AG.

Subgroup	N	Sensitivity (95% CI)	Specificity (95% CI)	DOR (95% CI)	AUC (95% CI)
Measurement of SPG					
Ratio of PGI:PGII	9	0.69 (0.52–0.83)	0.84 (0.68–0.93)	11.51 (6.14–21.56)	0.83 (0.80–0.86)
Combination of concentration of PGI and the ratio of PGI:PGII	3	0.79 (0.72–0.85)	0.89 (0.85–0.93)	24.64 (6.95–87.37)	0.87 (0.81–0.92)
Concentration of PGI	3	0.46 (0.38–0.54)	0.93 (0.91–0.95)	19.86 (0.86–456.91)	0.86 (0.52–1.00)
Concentration of PGII	1	Null	Null	Null	Null
Detection method					
ELISA	11	0.67 (0.65–0.69)	0.68 (0.65–0.70)	7.51 (4.96–11.37)	0.77 (0.75–0.80)
RIA	4	0.80 (0.75–0.85)	0.89 (0.87–0.91)	35.01 (7.31–167.66)	0.86 (0.69–0.96)
L-TIA	1	Null	Null	Null	Null
Study design					
Cohort	13	0.69 (0.67–0.71)	0.77 (0.75–0.78)	14.69 (8.33–25.91)	0.85 (0.80–0.91)
Case-control	3	0.62 (0.51–0.71)	0.80 (0.71–0.87)	12.40 (1.02–150.57)	0.82 (0.52–1.00)
Clearly defined inclusion and exclusion criteria					
Yes	10	0.69 (0.64–0.73)	0.67 (0.64–0.70)	8.77 (5.35–14.38)	0.81 (0.75–0.87)
No	6	0.69 (0.67–0.71)	0.86 (0.84–0.88)	30.35 (8.12–113.43)	0.90 (0.79–0.96)

Note: AUC, area under the summary receiver operating characteristic curve; DOR, diagnostic odds ratio; RIA, radio-immunity assay; ELISA, enzyme-linked immunosorbent assay; L-TIA, latex-enhanced turbidimetric immunoassay; CI, confidence interval

### Sensitivity analysis

We performed a sensitivity analysis to evaluate the effects of each individual study on the summary accuracy of SPG detection for GC and AG, as shown in Table 4 and Table 5. After each study was separately removed, the summary sensitivity, specificity, DOR and AUC ranges with 95% CIs were calculated. We found a relatively stable diagnostic accuracy of SPG detection for GC and AG in each group.

**Table 4:** Sensitivity analyses for the diagnostic accuracy of SPG for GC.

Study omitted	Sensitivity (95% CI)	Specificity(95% CI)	DOR(95% CI)	AUC (95% CI)
F Kitahara, 1999	0.68 (0.59–0.76)	0.73 (0.61–0.83)	5.80 (3.48–9.68)	0.75 (0.71–0.79)
Abraham M. Y. Nomura, 2005	0.71 (0.63–0.78)	0.71 (0.58–0.82)	6.16 (3.61–10.49)	0.77 (0.73–0.80)
Masahira Haneda, 2012	0.70 (0.61–0.77)	0.74 (0.61–0.83)	6.37 (3.79–10.69)	0.77 (0.73–0.80)
Ryousuke kikuchi, 2011	0.68 (0.59–0.76)	0.74 (0.60–0.83)	6.05 (3.55–10.30)	0.76 (0.72–0.79)
KENTARO SHIKATA, 2012	0.69 (0.60–0.77)	0.74 (0.62–0.83)	6.11 (3.59–10.38)	0.76 (0.72–0.80)
Rafael Lomba-Viana, 2012	0.69 (0.60–0.76)	0.75 (0.64–0.84)	6.48 (3.95–10.62)	0.77 (0.73–0.80)
Jung Mook Kang, 2008	0.70 (0.61–0.78)	0.74 (0.62–0.83)	6.59 (4.01–10.83)	0.77 (0.73–0.81)
Xiao-mei Zhang, 2014	0.69 (0.60–0.77)	0.71 (0.60–0.80)	5.39 (3.41–8.52)	0.75 (0.71–0.79)
SHIGETO MIZUNO, 2009	0.69 (0.60–0.77)	072 (0.60–0.82)	5.71 (3.45–9.47)	0.76 (0.72–0.79)
Yu-Yan Huang, 2013	0.66 (0.58–0.73)	0.76 (0.66–0.84)	6.12 (3.62–10.33)	0.75 (0.71–0.79)
Zhong-Lin Yu, 2008	0.70 (0.61–0.77)	0.73 (0.60–0.82)	6.16 (3.63–10.56)	0.77 (0.73–0.80)
Masaharu Yoshihara, 1998	0.67 (0.59–0.75)	0.76 (0.65–0.84)	6.38 (3.85–10.58)	0.76 (0.73–0.80)
F.-Y. CHANG, 1992	0.69 (0.60–0.77)	0.73 (0.60–0.82)	5.91 (3.50–9.96)	0.76 (0.72–0.80)
Metin Agkoc, 2010	0.68 (0.58–0.75)	0.71 (0.60–0.80)	5.15 (3.40–7.79)	0.74 (0.70–0.78)
Kazuo Aoki,1997	0.69 (0.60–0.77)	0.72 (0.60–0.82)	5.77 (3.46–9.62)	0.76 (0.72–0.79)

Note: AUC, area under the summary receiver operating characteristic curve; DOR, diagnostic odds ratio; CI, confidence interval

**Table 5:** Sensitivity analyses for the diagnostic accuracy of SPG for AG.

Study omitted	Sensitivity (95% CI)	Specificity (95% CI)	DOR (95% CI)	AUC (95% CI)
Manami Inoue,1998	0.68 (0.53–0.80)	0.90 (0.77–0.95)	17.00 (7.88–36.70)	0.85 (0.82–0.88)
F. Sitas, 1993	0.72 (0.59–0.82)	0.88 (0.75–0.95)	17.89 (8.47–37.79)	0.86 (0.82–0.88)
A. Oksanen, 2000	0.73 (0.61–0.82)	0.86 (0.74–0.92)	15.94 (7.70–31.37)	0.85 (0.82–0.88)
Cai-yun He, 2011	0.69 (0.54–0.81)	0.89 (0.77–0.95)	18.27 (8.61–38.76)	0.86 (0.83–0.89)
Diana Aulia, 2009	0.69 (0.54–0.81)	0.89 (0.79–0.95)	18.68 (9.26–37.69)	0.87 (0.83–0.89)
Metin Agkoc, 2010	0.67 (0.53–0.79)	0.87 (0.75–0.94)	13.56 (7.33–25.08)	0.84 (0.80–0.87)
Katsunori Iijima, 2009	0.71 (0.56–0.82)	0.87 (0.74–0.94)	16.42 (7.70–35.03)	0.86 (0.82–0.88)
Hyojin Chae, 2008	0.68 (0.53–0.80)	0.88 (0.75–0.95)	15.25 (7.36–31.61)	0.85 (0.81–0.87)
R. Sierra, 2006	0.67 (0.53–0.78)	0.90 (0.80–0.95)	17.32 (8.31–36.12)	0.86 (0.83–0.89)
Ma ´rio Dinis-Ribeiro, 2004	0.69 (0.55–0.81)	0.89 (0.77–0.95)	18.00 (8.42–38.47)	0.86 (0.83–0.89)
Kai Chun WU, 2004	0.67 (0.53–0.79)	0.89 (0.76–0.95)	15.88 (7.32–33.52)	0.84 (0.81–0.87)
N Broutet, 2003	0.70 (0.55–0.81)	0.89 (0.77–0.95)	18.17 (8.52–38.76)	0.86 (0.83–0.89)
Abbas Zoalfaghari, 2013	0.69 (0.54–0.81)	0.89 (0.77–0.95)	18.07 (8.51–38.34)	0.86 (0.83–0.89)
David Y Graham, 2006	0.68 (0.54–0.80)	0.89 (0.77–0.95)	17.43 (8.24–36.86)	0.86 (0.82–0.88)
M. Kekki, 1991	0.67 (0.53–0.79)	0.87 (0.74–0.94)	13.55 (7.14–25.74)	0.84 (0.80–0.86)
G. Nardone, 2005	0.71 (0.58–0.82)	0.86 (0.74–0.92)	14.75 (7.39–29.46)	0.85 (0.82–0.88)

Note: AUC, area under the summary receiver operating characteristic curve; DOR, diagnostic odds ratio; CI, confidence interval.

### Publication bias

**Fig 8:**
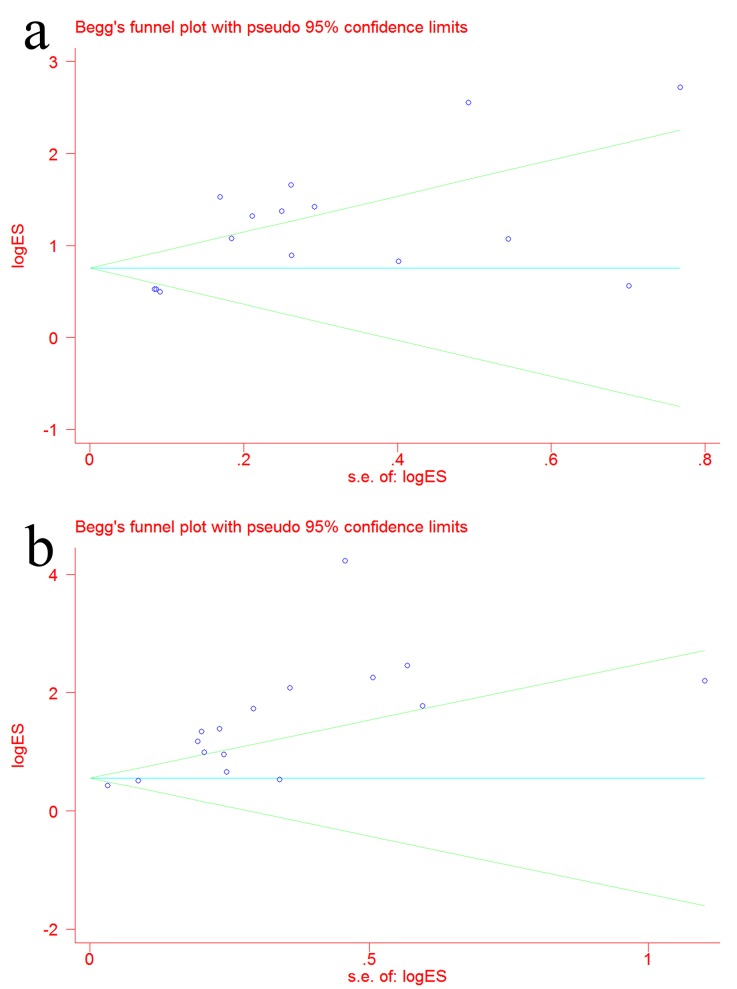
Begg’s funnel plot was constructed to demonstrate publication bias. (a) Begg’s funnel plot for GC; (b) Begg’s funnel plot for AG.

To analyze the publication bias of the included studies, Begg’s funnel plot was constructed. As shown in Fig 8, the P value was 0.002 for GC and <0.001 for AG, indicating potential publication bias among the studies.

## Discussion

GC was the world’s third leading cause of cancer mortality in 2012 and was responsible for 723,100 deaths [49, 50]. Korea, Japan, and China are among the areas with increased risk of GC[51]. Despite the decreased incidence rate of GC observed around the world, its prognosis remains poor. To effectively improve the survival rate of GC, improved large-scale screening tools for earlier diagnosis of GC and the identification of people at high risk for GC must be developed. Precancerous lesions of GC include AG, IM, and dysplasia, and it has been estimated that 0%–1.8%, 0%–10%, and 0%–73% of patients with AG, IM, and dysplasia, respectively, progress to GC annually[52]. Several noninvasive tests, including photofluorography, serum levels of PGI and PGII and *H*. *pylori* serology, are performed to screen for GC or precancerous lesions of GC. However, photofluorography has several disadvantages, such as X-ray exposure for individuals who are screened and low sensitivity in detecting early GC [10]. *H*. *pylori* serology is also not advantageous as a single screening modality because of its low specificity in distinguishing precancerous lesions [53]. SPG is a biomarker used to identify AG, and its potential utility in the diagnosis of GC has been demonstrated by numerous studies. Subsequently, cancer screening programs in Japan have accepted the measurement of SPG as a noninvasive screening test of GC. The measurement of SPG may detect AG or IM in a noninvasive manner, which is helpful to reduce the related morbidity and mortality of GC. In addition, the cost for the detection of a single cancer case by SPG is much less than that for conventional screening ($37,360 by conventional screening vs. $19,282 by SPG testing)[14]. However, only a few meta-analyses on the accuracy of SPG for predicting GC or precancerous lesions of GC are available. Our study performed a meta-analysis to clarify the diagnostic value of SPG.

The present study, including a total of 3,785 patients, identified a moderate capacity for SPG to detect GC and AG; the summary sensitivity and summary specificity for GC diagnosis were 0.69 (95% CI: 0.60–0.76) and 0.73 (95% CI: 0.62–0.82), respectively. Concurrently, the summary sensitivity and summary specificity for AG diagnosis were 0.69 (95% CI: 0.55–0.80) and 0.88 (95% CI: 0.77–0.94), respectively. The AUC values were calculated to evaluate the discriminating ability of this diagnostic method[54]. DOR combines sensitivity and specificity to assess diagnostic accuracy. The AUC and DOR of the SPG test for GC diagnosis were 0.76 (95% CI: 0.72–0.80) and 6.01 (95% CI: 3.69–9.79), respectively. For AG, the AUC and DOR were 0.85 (95% CI: 0.82–0.88) and 16.50 (95% CI: 8.18–33.28), respectively. A Fagan plot indicated that the use of SPG could moderately improve the GC and AG detection rate, confirming a moderate efficiency of SPG detection in GC and AG diagnosis. Nevertheless, we believe that SPG detection has a potentially significant role in GC mass screening, especially in the identification of populations at high risk for GC [25]. The study conducted by Jennifer M Yeh and colleagues suggested that targeting high-risk smokers for SPG screening might be a cost-effective strategy to reduce intestinal-type non-cardia GC mortality [55]. If combined with an additional GC screening method, such as serum MG7-Ag, serum gastrin-17, serum ghrelin and serum trefoil factor family 3 (TFF-3), the efficiency of GC screening could be improved. The combination of serum IgG anti- *H*. *pylori* antibody, gastrin, PG I and PG II was identified to be useful for predicting the presence of GC[56]. Susumu Aikou et al demonstrated that serum TFF-3 could be an effective marker of GC with sensitivity of 80.9% and specificity of 81.0%, while the combination of serum TFF-3 and SPG statistical significantly improved tumor detection as compared to TFF3 or SPG alone[57]. Zhigang Huang and colleagues also suggested that the combined testing of serum TFF-3 and SPG could farther improve the efficacy of GC screening [58]. In addition, Combinations of SPG, gastrin-17 and *H*. *pylori* antibody can also identify AG more effectively [59, 60]. The plasma levels of ghrelin was indicated to be correlated well with the serum levels of PGI as well as the PGI/II ratio in AG patients, suggesting that it could be an intriguing non-invasive marker for AG[61]. Inverse associations between ghrelin and GC was observed, suggesting a potential role for serum ghrelin as a biomarker of GC [62]. Based on the rapidly growing research area of proteomics, promising serum GC and AG biomarkers will hopefully be developed in the near future[63]. These studies may provoke more detailed investigations leading to identification of a panel of diagnostic serological markers applicable to GC surveillance programmes.

Substantial heterogeneity was noted in the interpretation of the results of the 11 included studies for GC: (1) Potential sources of heterogeneity derived from the different scales used for the patients were explored by meta-regression. Some studies focused on the screening value of SPG detection for GC, leading to the inclusion of fewer patients and more controls, whereas others focused on confirming the diagnostic value of SPG detection. Studies with smaller numbers of patients may have found a lower reliability of SPG as a diagnostic test. (2) 3 studies did not exhibit an appropriate interval between the reference standard and the index test. In this setting, if SPG was detected long before endoscopy, the patient's condition would progress. If patients with positive SPG detection received special treatment before undergoing endoscopy and biopsy, this condition might have interfered with the diagnostic test results. The unclear interval between SPG detection and endoscopic biopsy potentially constitutes a source of heterogeneity. (3) Two of the 15 included studies exclusively enrolled early GC patients, and the SPG concentrations of early GC patients might differ from those of advanced GC patients, thus constituting another potential source of heterogeneity. (4) Seven of the 15 included studies used RIA to detect SPG, 3 used ELISA, 3 used CLIA, and only 2 used L-TIA. These different detection methods could have generated different normal ranges and cut-off values of SPG. Although the present study indicated that SPG detection using ELISA exhibited an increased diagnostic accuracy, this difference might induce potential heterogeneity. For AG diagnosis, the study design, SPG detection method and measurement of SPG constitute significant sources of heterogeneity among the studies. Thirteen of 16 studies had cohort design, whereas 3 had case-control design. The different study designs may influence the diagnostic accuracy. Eleven of the 16 studies used ELISA to detect SPG, whereas 4 used RIA, and 1 used L-TIA. An excellent correlation was observed between ELISA and RIA for SPG detection[64], but the different methods yielded different cut-off values, which led to different diagnostic accuracies. Nine studies used the ratio of PGI:PGII as the measurement of SPG, 3 used both the concentration of PGI and the ratio of PGI:PGII, 3 used the concentration of PGI, and 1 used the concentration of PGII. PGI<70 ng/ml and PGI:PGII<3.0 are widely accepted as the cut-off points for GC screening in Japan[19], but the ratio of PGI:PGII has mostly been used in AG diagnosis.

Several potential limitations of the present study must be acknowledged. First, although a serum PGI concentration of<70 ng/ml and a PGI:II ratio of <3.0 have been widely accepted as the cut-off points in Japan, the included studies employed various SPG cut-off values and different SPG analytical technologies, and different studies exhibited different sensitivities and specificities. Despite this variation, we performed subgroup analyses and plotted the SROC curves to counteract the influence of various analytical technologies and different cut-off values; however, the potential for residual influence on the accuracy of the summary diagnostic parameters remains. Second, GC has different subtypes and different tumor sites, such as the intestinal and diffuse types as well as cardia and non-cardia carcinoma. Intestinal-type GC has no link to AG, so serological markers of gastric mucosal changes are minimal, but those changes are obvious in diffuse-type GC. Gastric cardia and non-cardia cancer are also different aetiologically, cardia cancers are made up from two distinct aetiologies, one related to *H*.*pylori* and AG (similar to non-cardia cancer), and the other related to gastro-oesophageal reflux disease (similar to oesophageal adenocarcinoma), SPG in patients with gastric cardia cancer may be different from patients with gastric non-cardia cancer. Due to the absence of sufficient data about subtypes of GC, the assessment of diagnostic value of SPG for GC detection is limited. In addition, serum levels of PGI and the ratio of PGI:II decreased more significantly in AG patients with IM than in AG patients without IM. *H*. *pylori* infection status can also affect the concentration of SPG in AG patients and GC patients. These unrecorded differences in the patients of the selected studies potentially contributed to the observed heterogeneity[65]. Because these details were not available, our ability to explore the source of heterogeneity was restricted. However, a more homogeneous analysis may have resulted in selection bias. Third, potential publication bias was observed among the included studies, suggesting that the diagnostic value of SPG in both GC and AG detection may be overestimated because of selective reporting. Studies with favorable results are more likely to be published. Fourth, some of the studies had a case-control design, which may be prone to overstating the accuracy of a diagnostic trial [66]. Fifth, even though increasing the detection rate of early GC could improve the overall prognosis[67], only 2 studies in the present analysis focused on the early detection of GC. There is a great need to conduct clinical trials to better identify the validity of SPG in the early detection of GC in the future. Sixth, most of the included studies for GC were based in Asian countries; only two eligible studies originated in Europe. This geographically limited distribution could also introduce sampling bias in GC diagnosis. It remains urgent to validate whether the value of SPG detection for GC screening applies to different populations worldwide.

In conclusion, SPG has the potential to play an important role in the identification of patients with AG, which are precancerous lesions of GC. SPG also constitutes a crucial component in GC screening for the susceptible population, although this biomarker only exhibits a moderate diagnostic value for identifying GC. Given the limitations and heterogeneity of the chosen studies, large-scale and well-designed prospective studies should be conducted to validate the clinical value of SPG in GC screening and the diagnosis of precancerous lesions of GC (AG, IM and dysplasia), especially in European and American

## Supporting Information

S1 Checklist PRISMA 2009 Checklist. (DOC)

S2 Checklist QUADAS-2 Checklist. (PDF)
