# Supplementary material for: Significance of Serum Pepsinogens as a Biomarker for Gastric Cancer and Atrophic Gastritis Screening: A Systematic Review and Meta-Analysis
Source: PLoS One. 2015 Nov 10;10(11):e0142080. doi: 10.1371/journal.pone.0142080 (PMC4640555; doi:10.1371/journal.pone.0142080)
Supplement: S2 Checklist — (PDF) [file pone.0142080.s002.pdf]

QUADAS-2

Phase 1: State the review question:

Patients (setting, intended use of index test, presentation, prior testing):
Index test(s):
Reference standard and target condition:

Phase 2: Draw a flow diagram for the primary study

Phase 3: Risk of bias and applicability judgments

QUADAS-2 is structured so that 4 key domains are each rated in terms of the risk of bias and the concern regarding applicability to the research question (as defined above). Each key domain has a set of signalling questions to help reach the judgments regarding bias and applicability.

DOMAIN 1: PATIENT SELECTION

A. Risk of Bias

Describe methods of patient selection:

- ❖ Was a consecutive or random sample of patients enrolled? Yes/No/Unclear
- ❖ Was a case-control design avoided? Yes/No/Unclear
- ❖ Did the study avoid inappropriate exclusions? Yes/No/Unclear

Could the selection of patients have introduced bias? RISK: LOW/HIGH/UNCLEAR

B. Concerns regarding applicability

Describe included patients (prior testing, presentation, intended use of index test and setting):

Is there concern that the included patients do not match the review question? CONCERN: LOW/HIGH/UNCLEAR

DOMAIN 2: INDEX TEST(S)

If more than one index test was used, please complete for each test.

A. Risk of Bias

Describe the index test and how it was conducted and interpreted:

- ❖ Were the index test results interpreted without knowledge of the results of the reference standard? Yes/No/Unclear
- ❖ If a threshold was used, was it pre-specified? Yes/No/Unclear

Could the conduct or interpretation of the index test have introduced bias? RISK: LOW /HIGH/UNCLEAR

B. Concerns regarding applicability

Is there concern that the index test, its conduct, or interpretation differ from the review question? CONCERN: LOW /HIGH/UNCLEAR

DOMAIN 3: REFERENCE STANDARD

A. Risk of Bias

Describe the reference standard and how it was conducted and interpreted:

- ❖ Is the reference standard likely to correctly classify the target condition? — Yes/No/Unclear
- ❖ Were the reference standard results interpreted without knowledge of the results of the index test? — Yes/No/Unclear

Could the reference standard, its conduct, or its interpretation have introduced bias? **RISK: LOW /HIGH/UNCLEAR**

B. Concerns regarding applicability

Is there concern that the target condition as defined by the reference standard does not match the review question? **CONCERN: LOW /HIGH/UNCLEAR**

DOMAIN 4: FLOW AND TIMING

A. Risk of Bias

Describe any patients who did not receive the index test(s) and/or reference standard or who were excluded from the 2x2 table (refer to flow diagram):

Describe the time interval and any interventions between index test(s) and reference standard:

- ❖ Was there an appropriate interval between index test(s) and reference standard? — Yes/No/Unclear
- ❖ Did all patients receive a reference standard? — Yes/No/Unclear
- ❖ Did patients receive the same reference standard? — Yes/No/Unclear
- ❖ Were all patients included in the analysis? — Yes/No/Unclear

Could the patient flow have introduced bias? **RISK: LOW /HIGH/UNCLEAR**
